# Exercise Preconditioning Attenuates the Response to Experimental Colitis and Modifies Composition of Gut Microbiota in Wild-Type Mice

**DOI:** 10.3390/life10090200

**Published:** 2020-09-14

**Authors:** Jinkyung Cho, Donghyun Kim, Hyunsik Kang

**Affiliations:** College of Sport Science, Sungkyunkwan University, Suwon 16419, Korea; lovebuffalo@gmail.com (J.C.); mola6861@skku.edu (D.K.)

**Keywords:** colitis, gut microbiota, high fat diet, inflammation, physical fitness

## Abstract

This study investigated the suppressive effect of exercise preconditioning against colitis induced by high-fat diet (HF) plus dextran sulfate sodium (DSS) in wild-type mice. Male mice (C57BL/6) aged 6 weeks were assigned to standard chow (SC, *n* = 10) or HF (*n* = 10) or HF followed by DSS (HF+DSS, *n* = 10) or exercise preconditioning (EX) followed by HF+DSS (EX+HF+DSS, *n* = 10) for a total of 15 weeks. After 12 weeks of dietary treatments and/or exercise preconditioning, mice in the DSS groups were subjected to administration of 2 cycles of 5-day DSS (2% w/v) with a 7-day interval between cycles. HF resulted in colitis symptoms and histological changes, infiltration of immunity cells, decreased gut barrier proteins, increased pro-inflammatory and chemotactic cytokines and decreased anti-inflammatory cytokine such as adiponectin, which deteriorated after administration of DSS. Exercise preconditioning alleviated HF+DSS-induced colitis and caused significant modifications in gut microbiota: decreased *Bacteroides vulgatus* (*p* = 0.050) and increased *Akkermansia muciniphila* (*p* = 0.050). The current findings suggest that exercise preconditioning attenuates the severity of HF+DSS-induced colitis in C57BL/6 mice.

## 1. Introduction

Inflammatory bowel disease (IBD) refers to immune disorders of the gastrointestinal (GI) tract, primarily Crohn’s disease and ulcerative colitis. Although North America and Europe have high prevalence of IBD, its incidence is also rising at an unprecedented rate in developing countries due to rapidly Westernized lifestyles [1].

The leading hypothesis for IBD is that an interaction between environmental and immune factors triggers disruption of intestinal barrier integrity, resulting in an exaggerated immune response to the commensal bacterial resident in the gut lumen [2] and chronic activation and inflammation in the intestinal immune system [3]. Pathologically, chronic inflammation is a common feature of IBD and obesity in both animals [4] and humans [5]. In this perspective, dysbiotic gut microbiota often observed in IBD patients [6] suggest that aberrations in gut microbiota composition associated with Westernized diets may also contribute to the pathogenesis of IBD [6].

In the meanwhile, it is well established that regular physical activity improves immune function [7] and decreases risk of intestinal inflammatory diseases including IBD [8]. In addition, exercise training improves gut barrier function by up-regulating heat shock protein (HSP) in the gut [9] and alleviates inflammatory responses in animal models of experimental colitis [10]. Exercise training modulates gut microbiota-enhancing symbiosis such that the host and microbe benefit mutually [11]. Together, those findings suggest that physical activity and/or modifications in gut microbiota may lead to therapeutic effects against the pathogenesis of colitis, although underlying mechanism(s) remained to be elucidated.

To the best of our knowledge, no previous study has investigated the suppressive effects of exercise preconditioning against colitis. In this study, we hypothesize that exercise preconditioning contributes to the host response to dextran sulfate sodium (DSS)-induced colitis in HF-fed mice, towards a balance between pro- and anti-inflammatory responses, leading to alleviation of the clinical symptoms of DSS-induced colitis. In addition, we also hypothesized that any alleviation in clinical symptoms is associated with exercise preconditioning-induced modifications in gut microbiota composition.

## 2. Materials and Methods

### 2.1. Animals

A total of 40 male C57BL/6 mice aged at 5 weeks were purchased from ORIENT BIO (Seongnam, Republic of Korea). Mice were housed in pairs at a pathogen-free animal care facility with controlled light (12:12 h light-dark cycle), humidity (50%) and temperature (20–23 °C) conditions and free access to food and tap water. The Sungkyunkwan University School of Medicine Institutional Animal Care and Use Committee (IACUC) in accordance with the American Association for Accreditation of Laboratory Animal Care (AAALAC) International Guidelines for animal experiments reviewed and approved the animal handling and procedures for this study (SKKUIACUC-17-5-1-1).

### 2.2. Experimental Design

**Figure 1:**
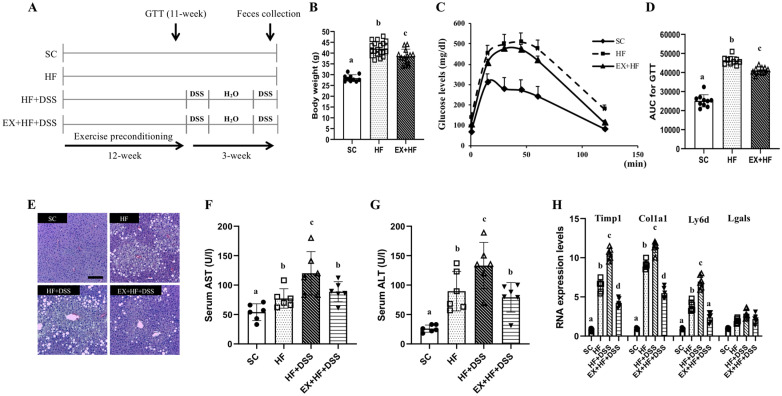
(**A**) Overall description of experimental design. (**B**–**H**). Exercise preconditioning alleviates metabolic complications associated with high fat diet (HF) plus dextran sulfate sodium (DSS)-induced colitis. (**B**) body weights (*n* = 12 mice per group) (**C**) glucose levels and (**D**) area under the curve (AUC) during glucose tolerance test (*n* = 10 mice per group) (**E**) liver hematoxylin and eosin (H&E) staining (magnification, ×10; scale bar, 20 μm, *n* = 5 mice per group) (**F**) serum aspartate aminotransferase (AST) (**G**) serum alanine aminotransferase (ALT) (*n* = 6 mice per group) and (**H**) hepatic mRNA expression of Timp1, Col1a1, Ly6d, and Lgals (*n* = 6 mice per group). GTT: glucose tolerance test; SC: standard chow; HF: high fat diet; EX: exercise preconditioning; DSS: dextran sulfate sodium. Data are presented as mean ± S.D. Different letters correspond to significant differences between groups. Statistical significances were tested with one-way analysis of variance.

Figure 1A represents overall study design. One week after adaptation to environment, mice aged 6 weeks were randomly assigned to standard chow (SC, *n* = 10) or high-fat diet (HF, *n* = 10) or HF plus dextran sulfate sodium (DSS) (HF+DSS, *n* = 10) or exercise preconditioning (EX) with HF+DSS (EX+HF+DSS, *n* = 10).

The SC and HF mice were fed with SC and HF, respectively, for a total of 15 weeks. The SC diet consisted of 10% fat, 70% carbohydrates, and 20% protein (kcal) and was provided in the form of regular pellets (Purina Mills, Seoul, Korea). The HF was prepared in small pellets of 60% fat (90% lard and 10% soybean oil), 20% carbohydrates, and 20% protein (kcal) (D12492 Research Diet, New Brunswick, NJ, USA). The HF used in this study is a well-established protocol to induce metabolic complications [10] along with pro-inflammatory and immune responses [11]. Body weights were measured weekly.

The EX+HF+DSS mice were trained on a motor-driven treadmill (Columbus Instruments, Inc., Columbus, OH, USA) with duration of 50 min per session and a frequency of 5 days per week for 12 weeks. Mice ran on the treadmill at zero inclination and a speed of 8 m/min for 5 min (warming up), 15 m/min for 40 min (main exercise at moderate intensity), and 8 m/min for 5 min (cool down) [12]. No electrical shock was used. In the meanwhile, the SC and HF mice were placed on the same treadmill without running for a matched period.

### 2.3. Glucose Tolerance Test

After 11 weeks of dietary treatments and/or exercise training, a glucose tolerance test (GTT) was performed with an intra-peritoneal injection (1.5 g/kg body weight) of glucose (Sigma-Aldrich, St. Louis, MI, USA) after 16-h fasting. Blood samples were collected from a cut at the tip of the tail before and 15, 30, 45, 60 and 120 min after glucose injection (a total of ~18 μL). Serum blood glucose was measured with One Touch II glucose meter (Lifescan, Johnson & Johnson, New Brunswick, NJ, USA). The linear trapezoid method was used to calculate area under the curve (AUC) for GTT. One week of recovery time was allowed prior to administration of DSS. We confirmed that all the mice were fully recovered from GTT.

### 2.4. DSS-Induced Colitis Model

After 12 weeks of dietary treatment and/or exercise training, the DSS-induced colitis model was established using a modified model from the previously reported protocol [13]. In brief, mice were given 2 cycles of 2% DSS (w/v) (reagent-grade, molecular weight of 36,000~50,000, MP Biochemical, cat. no. 02160110) in autoclaved drinking water for 5 days per cycle with a 14-day rest period between cycles and water ad libitum. Fresh DSS water was prepared daily. Control mice had free access to tap water. Clinical symptoms of colitis (e.g., weight loss, positive fecal hemoccult, and diarrhea) were monitored daily during and following administration of DSS.

### 2.5. Blood and Tissue Sampling

At the end of the experiments, blood was sampled from mice without food overnight. Blood collections were obtained from the abdominal vena cava and the hepatic portal vein just before sacrifice of mice under anesthesia with a mixture of Zoletil (40 mg/kg) and Rompun (5 mg/kg). Blood samples were centrifuged at 3000 rpm and 4 °C for 10 min and stored at −80 °C. Liver, colon, and spleen were carefully excised and flash frozen in liquid nitrogen. Prior to freezing, colon and spleen were photographed, and colon length and spleen weight were measured.

### 2.6. Biochemical Analyses

Serum concentrations of alanine aminotransferase (ALT) and aspartate Aminotransferase (AST) were measured with a Beckman DXC 800 analyzer (Beckman, Brea, CA, USA). Serum concentration of interleukin-6 (IL-6), interleukin-17A (IL-17A), monocyte chemoattractant protein 1 (MCP-1), chemokine growth-regulated protein (GRO)-alpha, transforming growth factor beta (TGF-β) and adiponectin were measured with ELISA kits (Abcam, Cambridge, UK).

### 2.7. Histopathology and Colitis Scoring

Tissues of colon and liver were fixed in 4% paraformaldehyde, embedded in paraffin, cut into 3 μm sections, and stained with hematoxylin and eosin (H&E). Morphological assessments of stained slides were performed with the Leica Qwin image analyzer system. Histological scoring was blindly performed by two researchers using the degree of surface epithelial loss, crypt destruction and inflammatory cell infiltration into the mucosa, scoring from 0 to 12 [14].

### 2.8. Real-Time PCR

Total RNA was extracted using RNA extraction kits (Applied Biosystems, Foster City, California, USA). Total RNA level was checked with a Nanodrop 2000 spectrophotometer (ThermoScientific, Brookfield, WI, USA). Fifty ng of total RNA was mixed with master mixture using TaqMan^®^ RNA-to-Ct™ 1-Step Kit and performed in an ABI prism 7500 real-time System (Applied Biosystems). Commercial FAM-labeled TaqMan probes (Applied Biosystems) were used as following: Timp1 (Mm01341361_m1), Col1a1 (Mm00801666_g1), Ly6d (Mm00521959_m1), Lgals (Mm00840285_m1), TLR4 (Mm00445273_m1), β-actin (Mm02619580_g1). Target gene expression was normalized against β-actin. Expression levels were calculated using the comparative cycle time method. All experiments were performed in triplicate.

### 2.9. Western Blot

Protein extracts from colon tissues were prepared by homogenization. Homogenates were centrifuged, and protein concentrations of supernatants were determined by the Bradford assay (Bio-Rad, Hercules, CA, USA). Western blot was performed as previously described [15]. The primary antibodies included mouse anti-occludin (ThermoScientific), rabbit anti-zonula occludens (ZO)-1 (ThermoScientific), rabbit anti-toll-like receptor 4 (TLR4) (ThermoScientific), and rabbit anti-heat shock protein (HSP) 70 (Cell Signaling, Beverly, MA, USA). The membrane was subsequently incubated with horseradish peroxidase (HRP) conjugated-secondary antibodies. Finally, blots were developed with a chemiluminescent HRP substrate kit (Millipore, Billerica, MA, USA). The intensity of the bands was determined by Image J version 1.8.0 software (National Institutes of Health, Bethesda, MD, USA) and normalized with β-actin (Bethyl Laboratories, Montgomery, TX, USA) densitometric values.

### 2.10. Flow Cytometry Analysis

For measurement of leukocytes in colon and blood, samples were stained with anti-CD45+, Ly6G, and Ly6C [16]. For mouse cell gating strategy, a live cell was stained with viability dye. CD45+ and CD11b+ were used as markers for total leukocytes and myeloid cell, respectively. Neutrophils were defined as CD45+CD11b+Ly6G+Ly6C+/low. CD45+CD11b+Ly6C+Ly6G– cells were defined as monocytes and excluded from neutrophil gating [16]. The cells were read by a FACSCanto II flow cytometer (BD Biosciences, San Jose, CA, USA) and analyzed using a FlowJo 7.6.5 analysis platform (Tree Star Inc., Ashland, OR, USA).

### 2.11. 16 S rRNA Gene and Metagenome Sequencing

Fecal samples were collected daily during the 14th week. Fecal bacterial DNA was extracted using the PowerMax^®^ *Soil DNA* Isolation Kit (MO BIO, Carlsbad, CA, USA). After extraction, DNA quantity and quality were checked with a PicoGreen^®^ dsDNA quantitation kit (Thermo Fisher Scientific, Seoul, Korea) and Nanodrop^TM^ (Thermo Fisher Scientific, Seoul, Korea), respectively. The DNA samples were then prepared according to Illumina 16S metagenomic sequencing library protocols for the amplification of the V3 and V4 region (519F-806R) with polymerase chain reaction (PCR). The bar-coded fusion primer sequences used for amplifications were 519F: 5′ CCTACGGGNGGCWGCAG 3′, 806R: 5′ GACTACHVGGGTATCTAATCC 3′. The final purified product was then quantified by quantitative PCR (qPCR) (KAPA Library Quantification kits for Illumina Sequencing platforms). The integrity of gDNA was checked with a LabChip GX HT DNA High Sensitivity Kit. (PerkinElmer, Waltham, MA, USA). Paired-end (2 × 300 bp) sequencing was performed with the MiSeq™ platform (Illumina, San Diego, CA, USA).

### 2.12. Metagenome Sequences Analysis

After sequencing MiSeq raw data, a FASTQ file was created using real-time analysis and bcl2fastq (v2.20.0.422). The paired-end data for each sample were assembled into a single sequence using FLASH (v1.2.11). Low-quality, ambiguous, and chimera sequences were removed using a CD-HIT-EST-based operational taxonomy unit (OTU) analysis program. Clustering of sequences with at least 97% sequence similarity yielded an OTU at the species level. The representative sequence of each OTU was produced with BLASTN (v.2.4.0) in the reference database (NCBI 16S Microbial). A Shannon index and inversed Simpson index were used to confirm species diversity and uniformity of microbial communities in environmental samples. Alpha diversity information was confirmed via a rarefaction curve and Chao1 value. Beta diversity between samples was obtained based on unweighted UniFrac Distance. Principal coordinates analysis (PCoA) was used to visualize the relationship between samples.

### 2.13. Statistical Analysis

Data were presented as mean ± standard derivation (SD). A Shapiro-Wilk normality test was performed to check normal distribution of data. One-way ANOVA, followed by Fisher’s Least Significant Difference (LSD) post-hoc test, if necessary, was used to compare any group differences with statistical significance of *p* = 0.05. A nonparametric t-test with 999 Monte-Carlo permutations was used to compare significant differences in alpha-diversity metrics (i.e., Chao1, Shannon, and Simpson). Nonparametric Kruskal-Wallis test using the Benjamini-Hochberg False Discovery Rate (FDR) correction was performed to test significant differences in relative abundance of taxa at *p* = 0.05. All analyses were carried out using SPSS-PC 25.0 (SPSS Inc., Chicago, IL, USA).

## 3. Results

### 3.1. Exercise Preconditioning Alleviates Metabolic Complications Associated with HF+DSS-induced Colitis

Figure 1B–D represent changes in weights and GTT after 12 weeks of dietary treatments and exercise preconditioning. As expected, chronic exposure to high-fat diet resulted in significant weight gains and higher AUC during GTT (as shown in HF vs. SC), which were alleviated by exercise preconditioning (as shown in EX+HF vs. HF). Figure 1E–H represent hepatic damage markers associated with dietary treatments and exercise preconditioning. HF resulted in significant elevations of serum AST and ALT enzymes and higher hepatic expression of Timp1, Colla1, and Ly6d mRNAs (as shown in HF vs. SC), which were further elevated by administration of DSS (as shown in HF+DSS vs. HF). On the other hand, exercise preconditioning attenuated HF+DSS-induced elevations of liver damage markers (as shown in EX+HF+DSS vs. HF+DSS).

### 3.2. Exercise Preconditioning Alleviates the Colitis Symptoms and Histological Changes Associated with HF+DSS-induced Colitis

**Figure 2:**
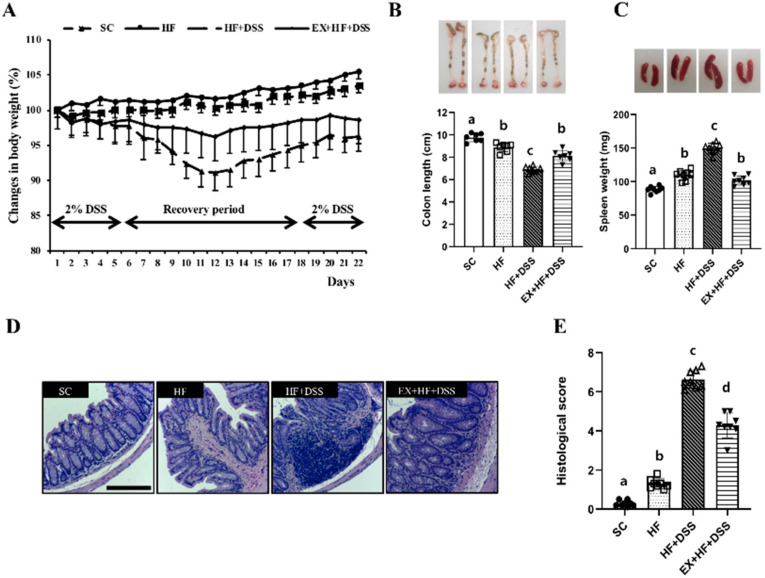
Exercise preconditioning alleviates the clinical symptoms associated with high-fat diet (HF) plus dextran sulfate sodium (DSS)-induced colitis. (**A**) changes in body weight (*n* = 12 mice per group) (**B**) colon length (*n* = 10 mice per group) (**C**) spleen weight (*n* = 10 mice per group) (**D**) colon hematoxylin and eosin (H&E) staining (*n* = 8 mice per group), and (**E**) colon histological scores (*n* = 8 mice per group). Data are presented as mean ± S.D. Different letters correspond to significant differences between groups. Statistical significances were tested with one-way analysis of variance.

Figure 2A–E represent clinical symptoms of colitis. HF resulted in significant weight gain (Figure 2A), shortening of colon length (Figure 2B), and enlargement of the spleen (Figure 2C), in conjunction with a significantly higher histological score in the colon (Figure 2D–E), as shown in HF vs. SC. Administration of DSS exacerbated HF-induced clinical symptoms and histological changes of colitis, as shown in HF+DSS vs. HF. On the other hand, exercise preconditioning alleviated the clinical symptoms and histological changes of HF+DSS-induced colitis, as shown in EX+HF+DSS vs. HF+DSS.

### 3.3. Exercise Preconditioning Prevents Altered Expression of Gut Barrier Proteins Associated with HF+DSS-induced Colitis

**Figure 3:**
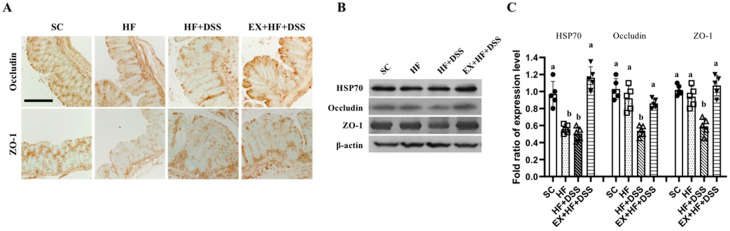
Exercise preconditioning prevents altered expression of tight junction markers associated with high-fat diet (HF) plus dextran sulfate sodium (DSS)-induced colitis. (**A**) Immunohistochemistry staining of colon (magnification, ×20; Scale bars, 20 μm; *n* = 6 mice per group) and (**B**,**C**) colonic protein levels of heat shock protein (HSP) 70, zonula occludens (ZO)-1 (*n* = 6 mice per group). Data are presented as mean ± S.D. Different letters correspond to significant differences between groups. Statistical significances were tested with one-way analysis of variance.

Figure 3A–C represent protein expression of occludin, ZO-1, and HSP70 in the colon. Chronic exposure to HF resulted in a significant decrease in HSP70 protein expression, as shown in HF vs. SC. Administration of DSS resulted in decreases in expression of ZO-1 and occluding proteins, as shown in HF+DSS vs. HF. On the other hand, exercise preconditioning prevented HF+DSS-induced alterations in the gut battier proteins expression, as shown in EX+HF+DSS vs. HF+DSS.

### 3.4. Exercise Training Alleviates Immune and Inflammatory Responses Associated with HF+DSS-induced Colitis

**Figure 4:**
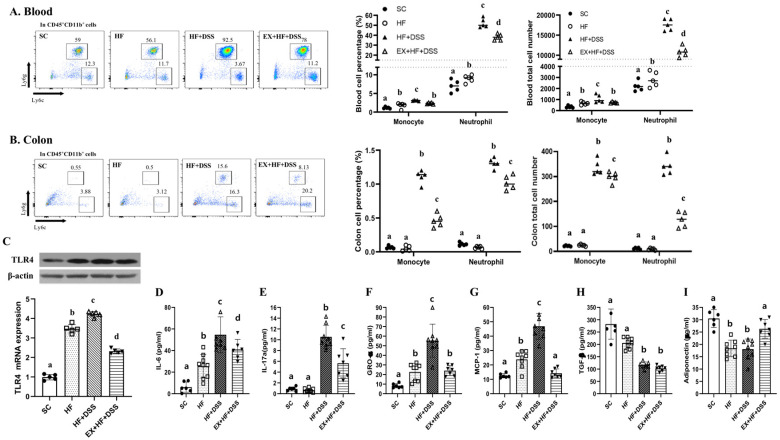
Exercise preconditioning suppresses increases in recruitment of immunity cells and pro-inflammatory and chemotactic responses associated with high-fat diet (HF) plus dextran sulfate sodium (DSS)-induced colitis; (**A**,**B**) neutrophil and monocytes in whole blood and colon (*n* = 5 mice per group) (**C**) toll-like receptor (TLR) 4 protein and mRNA levels and (**D**–**I**) serum levels of interleukin (IL)-6, IL-17A, growth-regulated oncogene (GRO)- α, monocyte chemoattractant protein (MCP)-1, transforming growth factor (TGF)-β, and adiponectin (*n* = 6–8 mice per group). Data are presented as mean ± S.D. Different letters correspond to significant differences between groups. Statistical significances were tested with one-way analysis of variance.

Figure 4A–C represents the total and percentages of immune cells in whole blood and colon. HF resulted in significantly higher numbers of circulating neutrophils and monocytes and increased levels of TLR4 gene and protein in the colon, with no significant changes in the immunity cells in the colon, as shown in HF vs. SC. Administration of DSS further increased levels of neutrophils and monocytes in the colon and blood in conjunction with further increased levels of TLR4 gene and protein in the colon, as shown in HF+DSS vs. HF. On the other hand, exercise preconditioning alleviated HF+DSS-induced increases of the immunity cells in the colon and blood and HF+DSS-induced increases of TLR4 gene and protein in the colon, as shown in EX+HF+DSS vs. HF+DSS.

Figure 4D–I represent pro- and anti-inflammatory and chemotactic cytokines. Chronic exposure to HF resulted in higher serum levels of IL-6, GRO-α, and MCP-1 and lower serum levels of adiponectin, with no significant changes in IL-17a and TGF-β, as shown in HF vs. SC, implying activation of inflammatory and chemotactic responses to HF. Administration of DSS exacerbated HF-induced activation of inflammatory and chemotactic responses, with no significant change in adiponectin, as shown in HF+DSS vs. HF. On the other hand, exercise preconditioning suppressed HF+DSS-induced up-regulation of inflammatory and chemotactic cytokines (i.e., IL-6, GRO-α, and MCP-1) and HF+DSS-induced down-regulation of adiponectin, as shown in EX+HF+DSS vs. HF+DSS.

### 3.5. Exercise Preconditioning Modifies Composition of Gut Microbiota

Table 1 represents alpha diversity parameters of gut microbiota, including observed OTUs, Chao1, Shannon’s and Simpson’s diversity indices, and rarefaction curves. HF resulted in higher values of OTUs (*p* = 0.022) and Shannon (*p* = 0.038), with no significance differences in Chao 1 and Simpson indices, as shown in HF vs. SC. Administration of DSS resulted in lower values of OTUs (*p* < 0.001), Chao1 (*p* = 0.003), Shannon (*p* = 0.001) and Simpson (*p* = 0.003), as shown in HF+DSS vs. HF. On the other hand, exercise preconditioning suppressed decreases in Shannon (*p* = 0.047) and Simpson (*p* = 0.028) associated with HF+DSS, with no such suppressive effects on OTUs and Chao1, as shown in EX+HF+DSS vs. HF+DSS.

**Figure 5:**
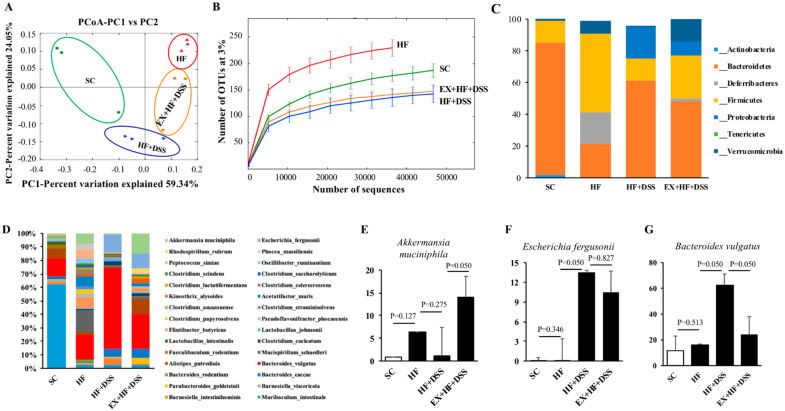
Exercise preconditioning increases diversity of microbiota in fecal samples. (**A**) Unweighted Unifrac Beta analysis indicates principal coordinates analysis (PCoA) - each point is colorized by treatment (*n* = 3 mice per group). (**B**) Diversity and (**C**) relative abundance of bacterial phylum levels. (**D**) Relative abundance of bacterial species levels (**E**) *Akkermansia muciniphila* (**F**) *Escherichia fergusonii* and (**G**) *Bacteroides vulgatus*.

Additionally, unweighted PCoA was conducted to compare the individual groups of mice and showed a clear separation of HF (red color) or HF+DSS (orange color) or EX+HF+DSS (blue color) from SC (green color). PC1 explained 59.34% of inter-sample variance and revealed a sharp distinction among the groups of mice (Figure 5A). Furthermore, the rarefaction curve showed significant differences in species richness among the groups of mice; SC vs. HF (*p* < 0.001), HF vs. HFD+DSS (*p* < 0.001), and HF+DSS vs. EX+HF+DSS (*p* = 0.032) (Figure 5B).

### 3.6. Exercise Preconditioning-induced Modifications in Gut Microbiota and Their Potential Associations with HF+DSS-induced Colitis

As illustrated in Figure 5C, analysis of fecal microbiota showed that Bacteroidetes (53.25%), Deferribacteres (5.51%), Firmicutes (26.25%), and Verrucomicrobia (5.97%) were the top four abundant phyla, accounting for 90.98% of the reads. At the phylum level, HF mice had a significantly higher Firmicutes to Bacteroidetes (F/B) ratio (*p* < 0.001) compared to SC mice. On the other hand, HF+DSS and EX+HF+DSS mice had significantly lower F/B ratios (*p* < 0.001 and *p* < 0.001, respectively) compared to HF mice. HF mice had also a higher abundance of Deferribacteres (*p* = 0.050) and a lower abundance of Actinobacteria (*p* = 0.050) compared to SC mice. HF+DSS mice had a lower abundance of Proteobacteria (*p* = 0.050) compared to HF mice. EX+HF+DSS mice had a significantly higher abundance of Verrucomicrobia (*p* = 0.050) compared to HF+DSS mice (Table 2).

As illustrated in Figure 5D, some noticeable modifications in gut microbiota were observed at the species level among the groups of mice. For example, HF+DSS mice had significantly higher *Bacteroides vulgatus* (*p* = 0.050) and *Escherichia fergusonii* (*p* = 0.050) compared to HF mice. On the other hand, EX+HF+DSS mice had significantly higher *Akkermansia muciniphila* (*p* = 0.050) and lower *Bacteroides vulgatus* (*p* = 0.050) compared to HF+DSS mice (Figure 5E–G).

## 4. Discussion

In this animal study, we showed that chronic exposure to HF resulted in metabolic complications and increased susceptibility to colitis in wild-type mice. The clinical symptoms of colitis (i.e., weight loss, shortened colon, and enlarged spleen) were exacerbated by mild administration of DSS. On the other hand, exercise preconditioning alleviated the severity of the clinical symptoms of colitis associated with HF+DSS treatment, as evidenced by alleviations in the colitis symptoms, altered expression of gut barrier proteins, infiltration of immunity cells into the colon and blood, and inflammatory and chemotactic responses. In particular, exercise preconditioning-induced alleviations of colitis pathological markers appear to be associated with symbiotic modifications in gut microbiota, although underlying mechanism(s) remain to be elucidated.

With respect to HF and/or HF+DSS, the current findings of the study are in line with previous studies reporting western diet-related susceptibility to colitis [17,18]. Gulhane et al. [17] showed that chronic exposure to HF resulted in impaired intestinal development in mice. Kim et al. [18] also reported that BALB/c mice fed 4 weeks of western-style diet had increased inflammatory responses in conjunction with increased infiltration of macrophages into the colon, which were exacerbated by administration of DSS. With respect to exercise preconditioning, the current findings are consistent with previous studies reporting the therapeutic effects of exercise training against pathogenesis of experimentally-induced colitis models by suppressing pro-inflammatory responses and simultaneously stimulating anti-inflammatory responses [19] in conjunction with enhanced gut barrier function and symbiotic modifications in gut microbiota [11]. In addition, exercise training-induced enhancement of the immune system was observed in both animals [19] and humans [20] with exaggerated or chronic inflammation. In particular, exercise training-induced symbiotic modifications in gut microbiota have been found in animal models of inflammatory disease models, such as colon cancer and IBD [21], ulcerative colitis [22], HF-induced obesity [23], and sepsis [24].

Several explanations can be given for exercise preconditioning-induced alleviation of clinical symptoms of this experimental colitis. First, exercise preconditioning-induced increase of HSP70 protein may enhance its protective function of gut epithelial cells and thereby minimize loss of gut barrier proteins (i.e., ZO-1 and occluding) under the colitis associated with HF+DSS treatments. In support of this notion, an experimental induction of HSP70 protein resulted in protection against the pathological progression of atrophic gastritis in Sprague-Dawley rats [25] and against Helicobacter pylori infection in normal rat gastric mucosal cells [26]. In this aspect, exercise and/or exercise training can function as a therapeutic means against colitis by inducing or activating HSPs in cardiac muscle, the liver, brain, and most notably skeletal muscle [27]. In addition, Luo et al. [28] showed that 7 consecutive days of moderate-intensity swimming attenuated repeated restraint stress-induced intestinal barrier dysfunction by stimulating antimicrobial responses in male Balb/c mice. Therefore, it seems reasonable to speculate that exercise preconditioning-induced activation of HSPs may protect the gut epithelium against oxidative stress and inflammation due to HF+DSS [28].

Second, exercise preconditioning may induce adaptations that help the host maintain a better balance between pro- and anti-inflammatory responses to colitis [29,30]. For example, Qin et al. [19] showed that 7 weeks of swimming ameliorated clinical symptoms of DSS-induced colitis by suppressing the production of pro-inflammatory and chemotactic cytokines in male Sprague-Dawley rats. Kasimay et al. [31] also showed that low-intensity exercise training may provide protection against oxidative colonic damage due to administration of DSS in wild-type rats. In the current study, we also found that exercise preconditioning decreased inflammatory responses along with decreased TLR4 protein expression, implying suppression of TLR4-dependent activation of pro-inflammatory response [32]. However, the functional meaning of exercise preconditioning-induced suppression of TLR4 expression observed in the current study should be confirmed with the assessment of TLR4 surface expression using flow cytometry.

In addition, the current findings also suggest that exercise preconditioning might enhance an anti-inflammatory response by suppressing HF+DSS-induced down-regulation of adiponectin. This is in an agreement with the findings of the study by Saxena et al. [33], which showed that moderate-intensity exercise training suppressed STAT3-mediated inflammation and apoptosis as well as infiltration of immune cells in the colon of adiponectin knock-out (APNKO) mice with DSS-induced colitis. In addition, aerobic exercise training increases antioxidant enzymes (glutathione peroxidase and catalase), anti-inflammatory cytokines (IL-10), and anti-apoptotic proteins (Bcl-2) in intestinal lymphocytes [29,30,34], while decreasing pro-apoptotic proteins such as caspase 3 and 7 as well as pro-inflammatory cytokine such as IL-17 and TNF-α [29,30].

Third, exercise/exercise training have been reported to be associated with symbiotic modifications in gut microbiota. In our study, the increased abundance of *Bacteroides vulgatus* and *Escherichia fergusonii* observed in HF+DSS mice compared to HF mice may reflect increased intestinal bacterial translocation via disrupted gut barrier and subsequent infiltration of acute inflammatory immune cells, exacerbating the colitis symptoms. In agreement with this, *Escherichia fergusonii* and *Bacteroides vulgatus* are implicated in bowel inflammation in animals [35] and humans [36] in IBD. On the other hand, exercise preconditioning resulted in increased diversity of gut microbiota along with an increase in *Akkermansia muciniphila* and a decrease in *B. vulgatus*. This is interesting because *Akkermansia muciniphila* has anti-inflammatory and immunostimulant properties and can improve gut barrier function, endotoxemia, and insulin sensitivity [37]. Similar to animal studies, exercise training-induced microbiota symbiosis has been reported from patients with metabolic diseases associated with gut barrier dysfunction and low grade inflammation [38]. Together, the current findings suggest that exercise preconditioning-induced symbiotic modifications in gut microbiota are associated with alleviated clinical symptoms of colitis associated HF+DSS treatments, although the mechanisms linking symbiotic gut microbiota to its protective effects against colitis need to be elucidated.

This study has some limitations. First, lack of positive controls (e.g., SC+DSS or EX+SC+DSS) in this study limits our interpretation regarding exercise training-induced alleviations of this experimental colitis. Second, the therapeutic effects of exercise preconditioning-induced modifications in gut microbiota against pathogenesis of colitis remain to be confirmed by using a germ free animal model in a cause-and-effect manner [39]. Third, assessing inflammatory responses and histological changes at multiple points rather than at end point only would provide a better picture of the unveiling of host responses to this experimental insult. Lastly, an objective measure of intestinal permeability, e.g., in vivo with a tracer flux assay or in vitro using chambers, would be necessary to elucidate a causal link between exercise training and gut barrier integrity.

## 5. Conclusions

In this animal study, we found that exercise preconditioning attenuated the clinical symptoms of experimentally induced colitis along with symbiotic modifications in gut microbiota in wild-type mice, implying a therapeutic role of promotion of physical fitness against the pathogenesis of colitis.

## Figures and Tables

**Table 1:** Alpha-diversity metrics of different mice groups (mean ± SD).

	Total Reads per Group	Coverage (%)	Observed OTUs	Chao1	Shannon	Simpson
SC	389582.000 ± 7299.597	0.990 ± 0.000	190.667 ± 9.838	229.720 ± 25.765	3.903 ± 0.247	0.847 ± 0.019
HF	363285.333 ± 4693.449	0.990 ± 0.000	233.333 ± 11.348	263.300 ± 12.401	4.870 ± 0.125	0.913 ± 0.009
HF+DSS	374658.000 ± 19939.972	0.990 ± 0.000	147.000 ± 11.358	160.053 ± 14.209	2.823 ± 0.194	0.683 ± 0.026
HF+EX+DSS	343162.000 ± 12509.490	0.990 ± 0.000	149.000 ± 9.866	166.183 ± 11.907	3.740 ± 0.437	0.830 ± 0.070

OTU: Operational taxonomic unit.

**Table 2:** Effects of exercise training on the composition of fecal microbiota at the family level.

Phylum	Family	Abundance (%)				Abundance Relative to SC (%)			*p* Value
		SC	HF	HF+DSS	HF+EX+DSS	HF	HF+DSS	HF+EX+DSS	
Actinobacteria	Bifidobacteriaceae	1.81	0.00	0.00	0.00	−1.81 *	−1.81 *	−1.81 *	0.027
Bacteroidetes	Bacteroidaceae	14.00	18.06	58.53	30.60	+4.06	+44.53 *	+16.60	0.026
Firmicutes	Streptococcaceae	0.01	1.04	0.26	0.30	+1.03 *	+0.25	+0.29	0.013
Firmicutes	Christensenellaceae	0.01	0.07	0.02	0.03	+0.06 *	+0.01	+0.02	0.006
Firmicutes	Clostridiaceae	1.99	0.65	0.00	0.02	−1.34 *	−1.99 *	−1.97 *	0.005
Firmicutes	Lachnospiraceae	0.69	18.67	3.85	8.84	+17.97 *	+3.15	+8.14 *	<0.001
Firmicutes	Oscillospiraceae	0.05	3.76	0.18	0.28	+3.71 *	+0.12	+0.22	0.001
Firmicutes	Peptococcaceae	0.38	6.44	0.78	1.02	+6.06 *	+0.40	+0.64	0.005
Firmicutes	Peptostreptococcaceae	0.01	0.11	0.86	0.18	+0.09	+0.85 *	+0.17	0.005
Firmicutes	Ruminococcaceae	1.44	5.53	0.56	1.75	+4.09 *	−0.88	+0.30	<0.001
Proteobacteria	Rhodospirillaceae	0.00	0.05	0.00	2.86	+0.05	0.00	+2.86 *	0.021

* *p* < 0.05 vs. SC.

## Abbreviations

HF	High fat
SC	Standard chow
DSS	Dextran sodium sulfate
EX	Exercise training
IBD	Inflammatory bowel disease
HSP	Heat shock protein
GTT	Glucose tolerance test
ALT	Alanine aminotransferase
AST	Aspartate aminotransferase
IL-6	Interleukin-6
MCP	Monocyte chemoattractant protein
GRO	Growth-regulated protein
ZO	Zonular occudens
TLR	Toll-like receptor
qPCR	Quantitative polymerase chain reaction
PCoA	Principal coordinates analysis
OUT	Operational taxonomic unit
FDR	False discovery rate
